# Postoperative arterial thromboembolism resulting in acute limb ischemia after staging surgery for endometrial carcinoma: A case report

**DOI:** 10.1016/j.gore.2022.100983

**Published:** 2022-05-02

**Authors:** Sarah Tounsi, Yingao Zhang, Sara Moufarrij, Anthony B. Costales

**Affiliations:** Baylor College of Medicine, Department of Obstetrics and Gynecology, Houston, TX 77030, United States; Baylor College of Medicine, Division of Gynecologic Oncology, Department of Obstetrics and Gynecology, Houston, TX 77030, United States

## 1 Introduction

Surgery is part of the mainstay of treatment for early-stage endometrial cancer (EMCA), and a minimally-invasive approach is a safe and proven surgical modality in the well-selected patient (Zullo et al., 2012). Comprehensive staging typically includes surgical resection of the uterus, cervix, adnexa, and a lymph node evaluation, either by sentinel or complete lymphadenectomy. When compared to laparotomy, minimally invasive surgery significantly decreases perioperative morbidity, rate of postoperative complications, length of hospital stays and is further compounded by the contemporary adoption of obtaining sentinel lymph node biopsies and targeted resections to replace complete lymphadenectomies (Walker et al., 2009, Rossi et al., 2017, Polan et al., 2019). Yuk and colleagues reported the incidence of venous thromboembolism (VTE) in women with endometrial cancer who have undergone either open or minimally invasive surgery ranges from 0.35 to 8% (Yuk et al., 2021). In another study, patients who underwent minimally invasive complete lymphadenectomy had a higher rate of VTE (2.1%) compared with sentinel lymphadenectomy (1.1%) and with patients who did not undergo lymphadenectomy (0.3%) (Polan et al., 2019).

Given the low incidence of VTE in this population, extended chemical VTE prophylaxis is not routinely recommended, even in individuals who fall into a higher risk category; high BMI and multiple medical comorbidities (Laskov et al., 2018, Carbajal-Mamani et al., 2021). Postoperative arterial thrombi are exceedingly rare after laparoscopic surgery and are only noted in scant case reports, mainly in patients with preexisting hematologic disorders, severe arteriosclerotic disease, or prior radiation therapy. Specifically in patients with gynecological cancer, there are less than 10 reported cases of acute postoperative arterial thromboembolic events in the current literature (Levenback et al., 1996, Nakamura et al., 2008, Ishikawa et al., 2020, Hamilton and Robinson, 1996) (Table 1).

**Table 1:** Reported cases of postoperative arterial thromboembolic events following surgery for gynecologic cancer.

Author	Year	Age	BMI	Diagnosis	Surgery	Chemotherapy	Lesion	POD	Treatment	Compartment syndrome?
Hamilton & Robinson (Ishikawa et al., 2020^)^	1996	58	n/a	IIIC tubal carcinoma	Primary cytoreduction (open)	Cisplatin, cyclophosphamide	NS (R hand and wrist)	20	Amputation	No
	1996	78	n/a	Vulvar carcinoma	Radical excision, superficial inguinal lymphadenectomy	n/a	Microemboli (L toe, R digits)	16	Amputation	No
	1996	n/a	n/a	IIIC ovarian carcinoma	Primary cytoreduction (open)	n/a	NS (R foot)	2	Therapeutic anticoagulation	No
Nakamura, et al. (Levenback et al., 1996)	2008	34	22	IIIC endometrial carcinoma	TAH, BSO, pelvic and para-aortic lymphadenectomy	n/a	L common iliac	1	Thrombectomy	Yes
Yeon, et al. (Creager et al., 2012)	2017	37	22	IB2 cervical carcinoma	TLH, BSO, pelvic and para-aortic lymphadenectomy	n/a	L external iliac	1	Thrombectomy	Yes
Ishikawa, et al. (Nakamura et al., 2008)	2020	53	n/a	IA endometrial carcinoma	TLH, BSO, pelvic lymphadenectomy	n/a	R external iliac*	1	Thrombectomy	No
Tounsi et al.	2021	62	42	IB endometrial carcinoma	TLH, BSO, sentinel lymphadenectomy	n/a	R external iliac, R popliteal	4	Thrombectomy	No

*This patient’s primary surgery was complicated by an intraoperative 2 mm external iliac artery laceration during laparoscopy.

We present the case of a patient with acute limb ischemia secondary to an arterial thrombus in the immediate postoperative period after an uncomplicated laparoscopic surgical staging for a stage IB grade 2 endometrioid EMCA.

## 2 Case report

The patient is a 63-year-old woman, gravida 4 para 2, with a past medical history significant for hypertension well controlled on Lisinopril, type 2 diabetes (HbA1 6.4%) controlled on Metformin, and class III obesity (BMI 42), who first presented with abnormal uterine bleeding, and was found to have FIGO grade 2 endometrioid EMCA on an endometrial biopsy. Pre-operative abdomen and pelvis computer tomography was performed that was only remarkable for a multi-fibroid uterus. She denied a history of bleeding or clotting disorders and did not have any significant cardiac or atherosclerotic disease. Her family history was unremarkable. Her past surgical history was pertinent for an exploratory laparotomy via a supra-umbilical horizontal incision after a trauma twenty years prior.

Six weeks after her initial diagnosis, she underwent an uncomplicated total laparoscopic hysterectomy, bilateral salpingo-oophorectomy, and bilateral sentinel lymph node mapping and biopsy. She received 5,000 units of unfractionated heparin subcutaneously for peri-operative prophylaxis 30 min prior to surgical incision. Total surgical time including being in dorsal lithotomy position was 123 min. She underwent routine postoperative recovery and was discharged home on the same day.

On postoperative day (POD) 1 she presented to the emergency room for sudden onset right foot pain associated with paresthesia and cold sensation. Bilateral lower extremity pulses were palpable, and the rest of the physical exam was found to be normal. Her labs were only significant for a magnesium level of 1.8 mg/dL, which was repleted orally. Her pain improved spontaneously without intervention, and she was discharged from the ED in stable condition.

She presented to the emergency room again on POD 3 for worsening pain and paresthesia. Her physical exam was significant for new right calf tenderness on palpation, but otherwise remained unremarkable with palpable and equal peripheral pulses in both lower extremities. A lower extremity venous doppler was performed with no evidence of deep venous thrombosis. Labs were not collected during that visit. She was given one dose of 15 mg ketorolac intravenously and 10 mg cyclobenzaprine PO with some improvement in her pain. Her vital signs, electrolytes and creatinine were within normal limits, and she was discharge in stable condition.

**Fig. 1:**
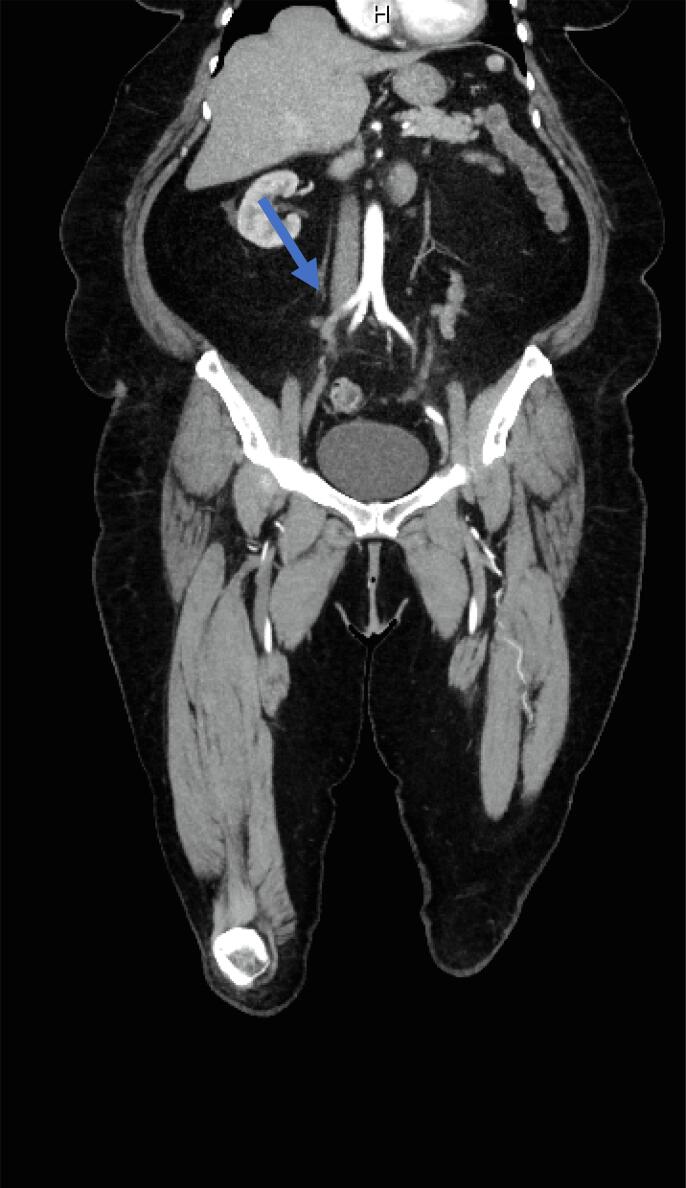

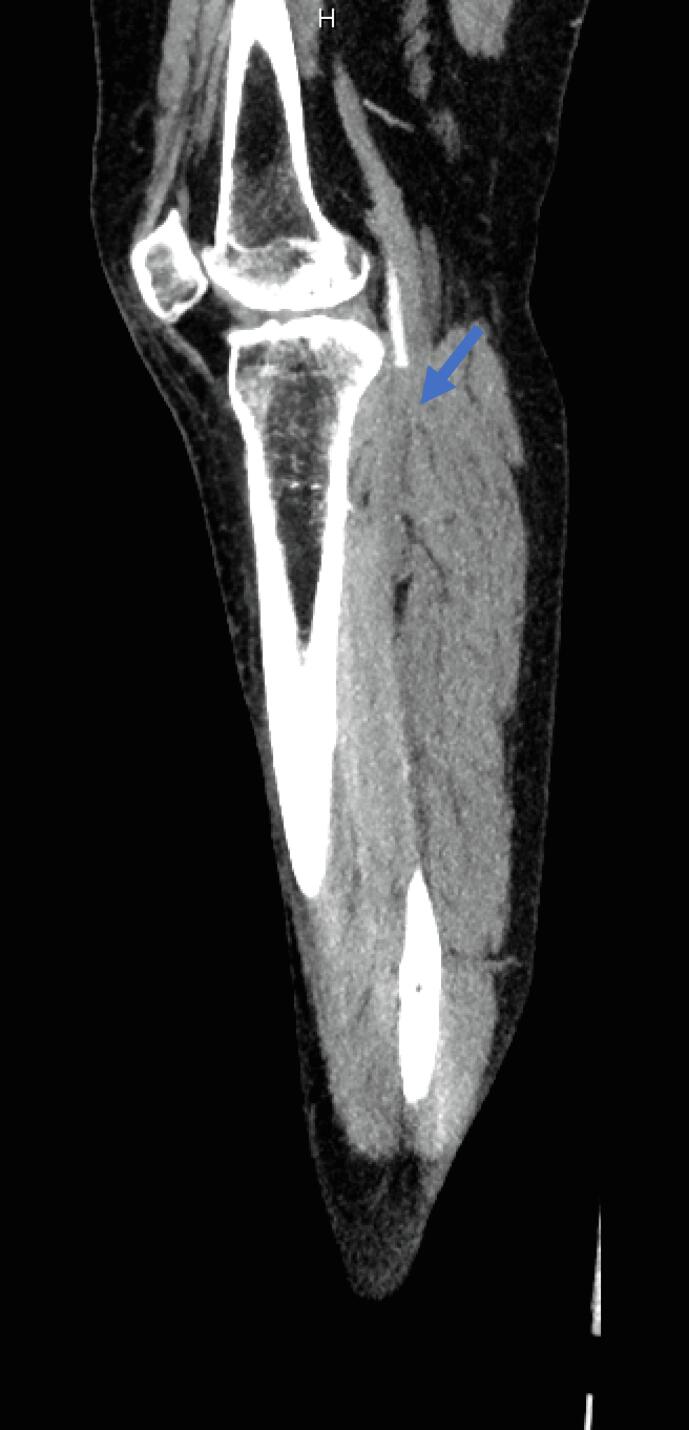

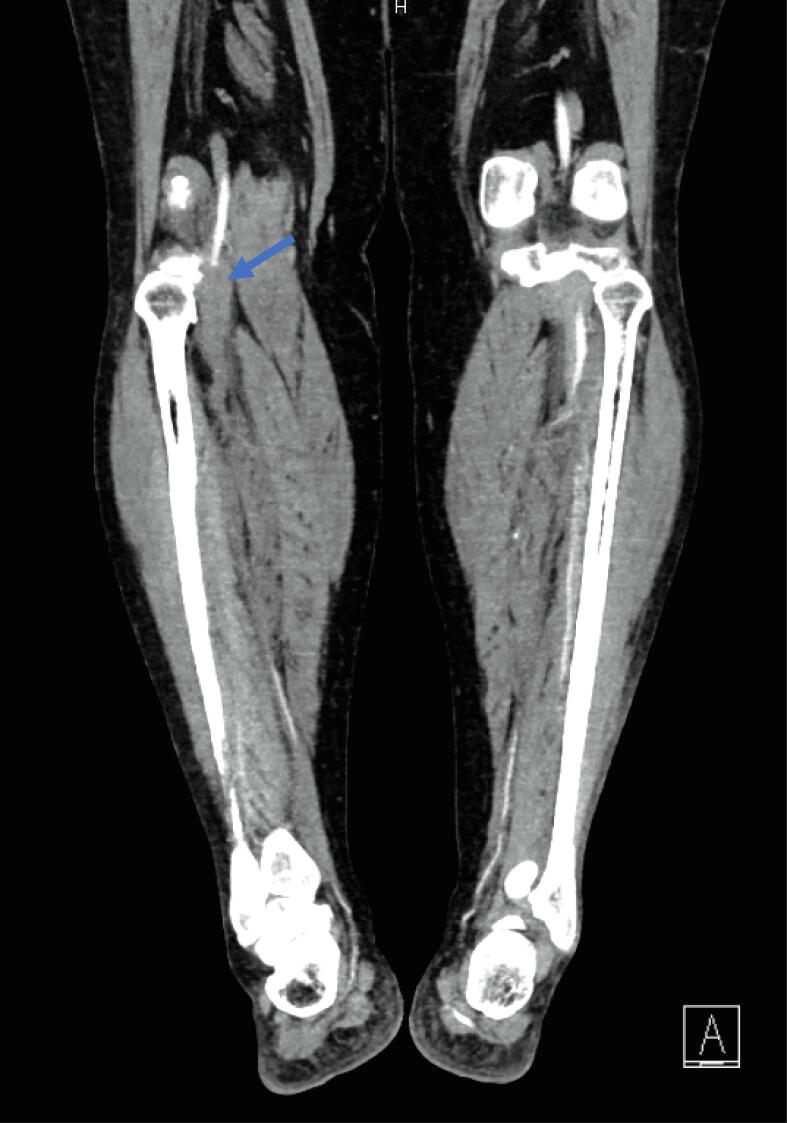
CTA images at patient presentation confirming arterial thromboembol.

On POD 4, she presented once more to the emergency room for worsening pain in her right leg. On presentation, the right dorsalis pedis (DP) and posterior tibial (PT) pulses were not palpable, and absent flow was confirmed by bedside doppler. A computed tomography angiography (CTA) showed a thrombus extending 10 cm from the right external iliac artery down into the femoral artery, as well as an 8 cm thrombus in the right popliteal artery, confirming the diagnosis of an acute arterial thromboembolism (Fig. 1). The patient was immediately started on an unfractionated heparin infusion and underwent an emergent right femoral artery cut down with a Fogarty® balloon thrombectomy of the right common iliac, external iliac, and superficial femoral arteries with primary closure of the arteriotomy. She was transferred to the surgical intensive care unit for hourly neurovascular checks and close monitoring for compartment syndrome. Coagulopathy work up was not performed during that admission. Approximately 5 h following thrombectomy, the right DP and PT pulses were lost. She then underwent an urgent external iliac artery endovascular stent placement, right anterior tibial artery Fogarty® balloon thrombectomy and right anterior tibial arteriotomy repair with bovine pericardial patch balloon angioplasty. She recovered well postoperatively and was transitioned from an unfractionated heparin infusion to weight-based low molecular weight heparin on hospital day 2. The remainder of her hospital course was uncomplicated, and she was discharged home on hospital day 7 on apixaban 5 mg twice daily, with plan to continue for at least 6 months. At point of last contact, she was recovering well without symptoms. Her final diagnosis was stage IB grade 2 endometrioid endometrial adenocarcinoma.

## 3 Discussion

Given its rarity, the prevalence of arterial thrombosis leading to acute limb ischemia in the immediate postoperative period following EMCA is not reported in the literature. It is often challenging to identify the etiology and describe the pathogenic mechanism involved. The classic presentation of patients with acute arterial occlusion without underlying occlusive vascular disease includes pain, pulselessness, pallor, poikilothermia, paresthesia and finally paralysis (Creager et al., 2012). Risk factors discussed in the literature include but are not limited to atherosclerotic disease, embolism from the heart, prolonged surgical positioning and trendelenburg, intravascular dehydration, hyper-coagulopathy, dissection, and vascular trauma^13^. In the medical literature, 9 case reports have previously described pelvic surgeries complicated by acute limb ischemia, 6 of which were in the setting of gynecologic malignancies (Sahara et al., 2019, Yeon et al., 2017, Nakamura et al., 2008, Ishikawa et al., 2020, Hamilton and Robinson, 1996); (Table 1).

The cases presented by Nakamura et al. and Yeon et al. involved extended operative times (>6 h) in lithotomy which was hypothesized to contribute to the occurrence of postoperative arterial thrombosis. The addition of head-down tilt during a case in lithotomy has also been proposed as a possible contributory factor by significantly decreasing lower limb perfusion intraoperatively (Horgan et al., 1999). The patient presented by Ishikawa et al underwent a shorter laparoscopic surgery (4 h) but was complicated by a tearing injury of the right external iliac artery wall during lymphadenectomy, which led to postoperative thrombus formation at the site on injury, requiring surgical excision of the injured arterial segment and primary vessel anastomosis. The authors attributed this complication to utilization of extended angio-pressure and fibrin sealant for hemostasis of the initial injury, and instead suggested a primary closure by suture in future cases. In our case, the surgery was relatively short (123 min) and uncomplicated without evidence or suspicion for occult vascular injuries.

While the patient in question was similarly positioned intraoperatively, it remains unclear whether this was the sole cause of the postoperative arterial thrombosis formation. Furthermore, while our patient did not have a previous diagnosis of atherosclerosis, her CTA showed signs of early disease with mild calcifications noted in common iliac arteries. She also had a significant medical history of hypertension, diabetes and obesity as well as the diagnosis of EMCA, all of which are known to increase the risk of both arterial and venous thrombosis formation (Yeon et al., 2017). Part of the host immune response to tumor cells include global cytokine upregulation, which can induce a hypercoagulable state within the vascular endothelium.

## 4 Conclusion

As previously mentioned, acute limb ischemia secondary to arterial thrombi in the immediate postoperative period is extremely rare and not clearly understood. In analyzing the case at hand, we noticed a delay in timely diagnosis as the patient presented to the emergency department three separate times with worsening pain. High clinical suspicion for acute limb ischemia postoperatively is needed for prompt diagnosis and adequate treatment. Thorough clinical evaluation should include assessment of limb color, temperature, pulses, and motor and sensory function (Creager et al., 2012). Thrombophilic work-up and obtaining an echocardiogram can also be considered after stabilizing the patient.

Future considerations for assessing patients at high risk for developing arterial thromboses can include evaluating whether ankle brachial pressure index needs to be routinely performed during pre-operative evaluation in patients with risk factors for atherosclerosis or in those with an existing diagnosis (Sahara et al., 2019). Another consideration can be using invasive monitoring of pedal blood pressure or pulse oximetry on lower extremities in high-risk patients to assess intraoperative decrease in lower extremity perfusion (Sahara et al., 2019). However given the rarity of such complication, routinely performing such evaluations may not be cost or time effective. Continuing to report similar cases in the medical literature is crucial to identifying the rate at which this morbidity is found in these patients.

## Declaration of Competing Interest

The authors declare that they have no known competing financial interests or personal relationships that could have appeared to influence the work reported in this paper.
